# Simulator training and residents’ first laparoscopic hysterectomy: a randomized controlled trial

**DOI:** 10.1007/s00464-019-07270-3

**Published:** 2019-11-25

**Authors:** Ewa Jokinen, Tomi S. Mikkola, Päivi Härkki

**Affiliations:** Obstetrics and Gynecology, University of Helsinki and Helsinki University Hospital, Haartmaninkatu 2, P.O. Box 140, 00029 HUS Helsinki, Finland

**Keywords:** Surgical education, Resident education, Virtual reality simulator, OSATS, VAS

## Abstract

**Background:**

Hysterectomy rates are decreasing in many countries, and virtual reality simulators bring new opportunities into residents’ surgical education. The objective of this study was to evaluate the effect of training in laparoscopic hysterectomy module with virtual reality simulator on surgical outcomes among residents performing their first laparoscopic hysterectomy.

**Methods:**

This randomized study was carried out at the Department of Obstetrics and Gynecology in Helsinki University Hospital and Hyvinkää Hospital. We recruited twenty residents and randomly signed half of them to train ten times with the laparoscopic hysterectomy module on a virtual reality simulator, while the rest represented the control group. Their first laparoscopic hysterectomy was video recorded and assessed later by using the Objective Structured Assessment of Technical Skills (OSATS) forms and Visual Analog Scale (VAS). The scores and surgical outcomes were compared between the groups.

**Results:**

The mean OSATS score for the Global Rating Scale (GRS) was 17.0 (SD 3.1) in the intervention group and 11.2 (SD 2.4) in the control group (*p* = 0.002). The mean procedure-specific OSATS score was 20.0 (SD 3.3) and 16.0 (SD 2.8) (*p* = 0.012), and the mean VAS score was 55.0 (SD 14.8) and 29.9 (SD 14.9) (*p* = 0.001). Operative time was 144 min in the intervention group and 165 min in the control group, but the difference did not reach statistical significance (*p* = 0.205). There were no differences between the groups in blood loss or direct complications.

**Conclusion:**

Residents training with a virtual reality simulator prior to the first laparoscopic hysterectomy seem to perform better in the actual live operation. Thus, a virtual reality simulator hysterectomy module could be considered as a part of laparoscopic training curriculum.

Although the number of live operations in residents' training has diminished [1], technology offers new solutions for surgical training in the form of lap trainers and virtual reality simulators [2–5]. With these training tools, it is possible to acquire fundamental laparoscopic skills [2], and they may be used as a part of the procedural training curriculum [6], as well. Though procedural skills have been proven to be transferred into the operating room after virtual reality simulator modules in laparoscopic cholecystectomy [7], in laparoscopic salpingectomy [8], and in cataract surgery [9], no data exists on the effect of virtual reality simulators on advanced major surgical procedures [10].

In gynecology, hysterectomy is a major benign surgery and it is simultaneously one of the most common gynecological procedures [11]. In this study, our aim was to evaluate the effect of training with the laparoscopic hysterectomy module on a virtual reality simulator on a resident's first laparoscopic hysterectomy as a first surgeon.

## Materials and methods

For this interventional and blinded study, 20 residents were enrolled between June 2013 and March 2016. The participants came from Helsinki University Hospital and Hyvinkää Hospital. All residents in gynecological surgery rotation with experience in laparoscopic diagnostic and adnexal surgery as a first surgeon, as well as assisting in more advanced laparoscopic procedures, were invited. None of the residents fulfilling the criteria declined to participate. Laparoscopic hysterectomy as a first surgeon or training with hysterectomy module with a virtual reality simulator were exclusion criteria. Participants were randomized using sealed envelopes into two equal size intervention and control groups by a research assistant outside the study.

Participant demographics were collected by questionnaires. They included age, experience in obstetrics and gynecology and in general surgery, experience in diagnostic and adnexal surgery as a first surgeon, and ongoing or past video game and musical instrument playing habits. Patient- and surgery-related data were collected from the medical records. They included age, body mass index, previous abdominal surgery, Cesarean sections and deliveries, weight of the removed uterus, concomitant adnexal surgery, operating time, blood loss, and complications.

All participants in the intervention and control group did the web-based theoretical course ‘Basics in gynecological laparoscopy’ [12], and trained five times each of the nine basic skill tasks in the same virtual reality simulator (LAP Mentor, Simbionix Corporation, Cleveland, Ohio, USA). Thus, all participants did the same intervention that was used in our recent study to evaluate its effect on residents’ first operative laparoscopy [13]. These practice sessions were automatically recorded and were used to assess the technical skill level in the beginning of the study. A composite score [14] was calculated for each task to standardize different dimensions of the tasks, and scores were normalized meaning that the mean performance had a score of 100, better performances scored > 100, and worse performances < 100. We weighted each dimension equally. Simultaneously, all participants took part in the standard clinical education at the wards with clinical lectures.

The intervention group trained ten times with the hysterectomy module without the guidance function in virtual reality simulator within 1 month before the surgery. The module was introduced before starting the training program. The rehearsal has a standardized surgical case with normal size uterus, and it starts with setting the camera on place and ends with colpotomy. The procedure is done following the standard steps introduced in the web material [12]. All practice sessions were automatically recorded, and all the recorded parameters were analyzed for learning curves. These parameters included total procedure time, idle time (total time that the moving instrument is not touching the tissue), total path length of instruments, total number of movements of the instruments, respect for tissue, and venous and organ injuries. Also for each participant, a composite score weighting each dimension equally was calculated for laparoscopic hysterectomy module.

The participants’ first laparoscopic hysterectomy as a first surgeon was a video recorded for later evaluation. In every operation, the first assistant was a senior doctor and the second assistant was a scrub nurse. If necessary, the assisting doctor instructed and directed the resident as is the norm in apprentice model.

In this study, the surgical recordings of both the intervention and the control group were assessed by two of the authors (P.H. and E.J.) who were blinded for the surgent and the study group. To assess overall management, we used the Visual Analog Scale (VAS) and a young specialist’s level as a reference. This refers to a skill level where the operator is able to perform independently a basic laparoscopic hysterectomy. To assess basic technical skills, we used the Objective Structured Assessment of Technical Skills (OSATS) form for Global Rating Skills (GRS) [15] and procedure-specific LH-OSATS to assess skills in laparoscopic hysterectomy.

**Fig. 1:**
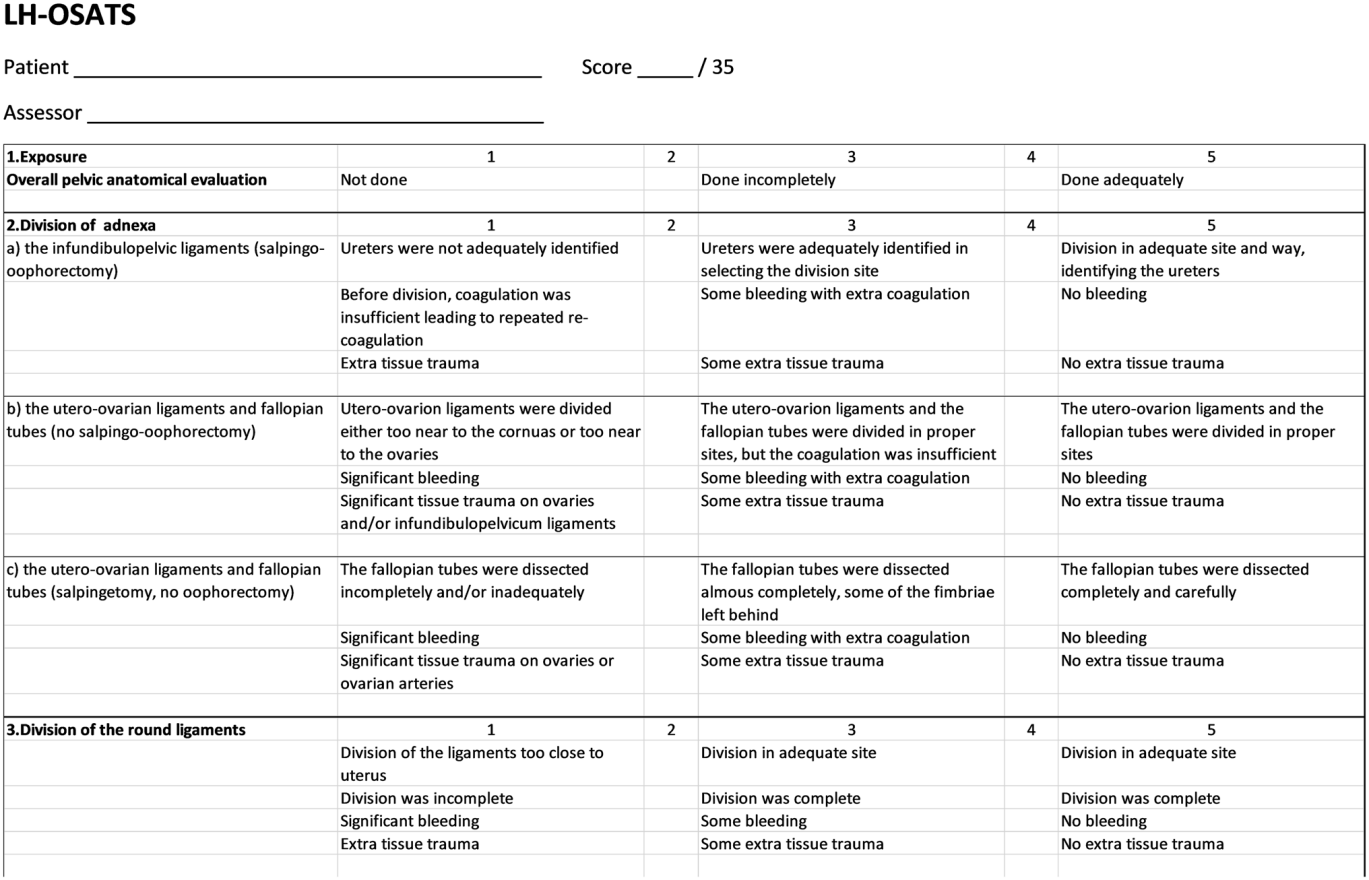

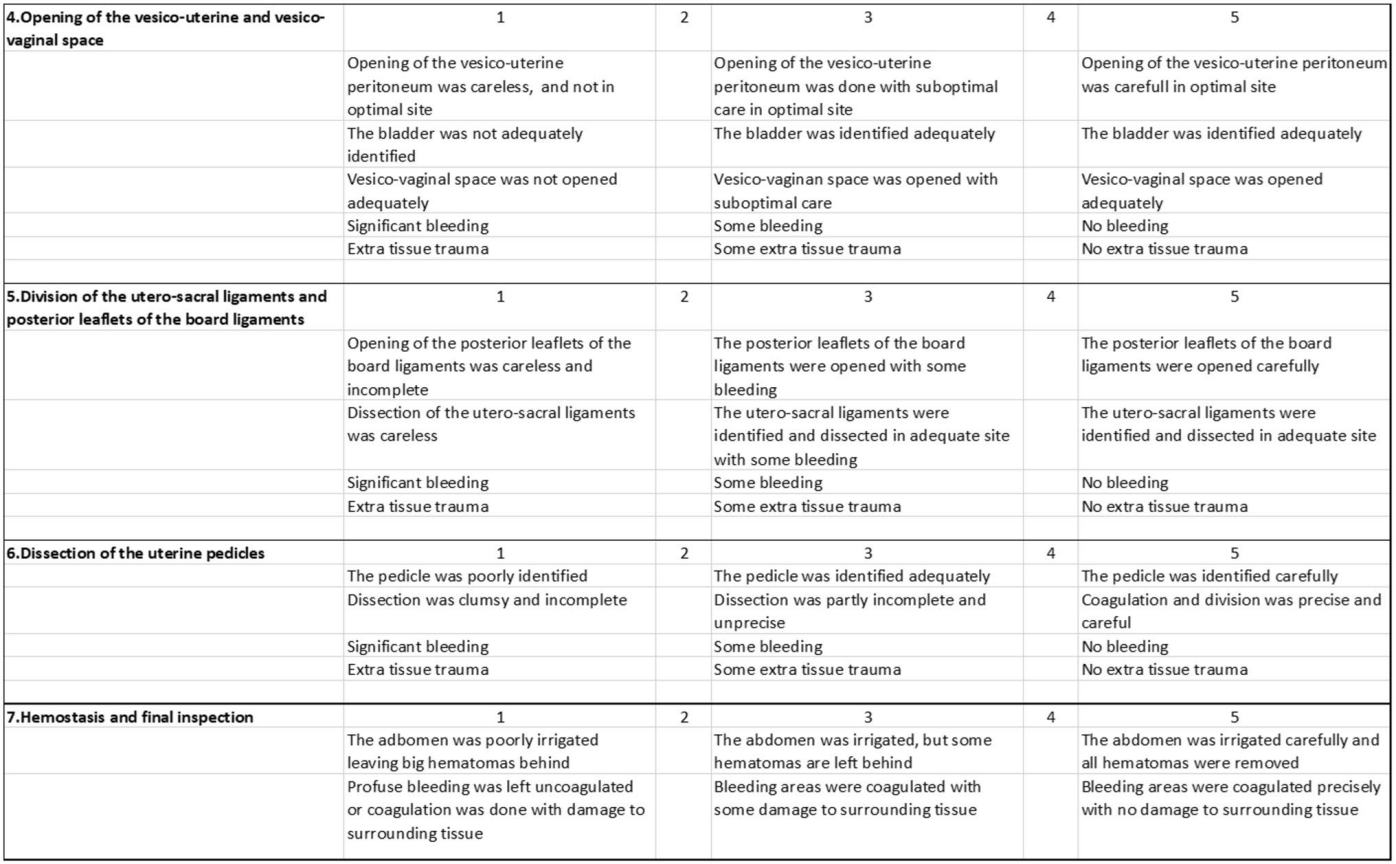
Procedure-specific OSATS form for laparoscopic hysterectomy

To assess procedure-specific skills, we developed the OSATS form for laparoscopic hysterectomy (LH-OSATS) (Fig. 1) with seven operational core steps including exposure, division of adnexa, division of round ligaments, opening of vesico-uterine and vesico-vaginal space, division of utero-sacral ligaments and posterior leaflets of board ligaments, division of uterine pedicles, and hemostasis and final inspection. For the LH-OSATS form construct validation study, we recorded 27 laparoscopic hysterectomies of which nine were operated by residents, nine by young specialists, and nine by experts. Those recordings were assessed by two of the authors (P.H. and E.J.) who were blinded for the operator. The OSATS scores between the three groups were compared.

As primary outcome measures, we used both OSATS forms giving scores 13–65 (6–30 from the GRS and 7–35 from the LH-OSATS) and the VAS giving scores 0–100. Before the assessment, the assessors made a mutual understanding of the use of the forms. Secondary outcome measures included operating time, blood loss, and direct complications.

We based our power calculations on OSATS scores. We assumed that the effect size of training on the hysterectomy module would be at least at the same level as training on the salpingectomy module in a virtual reality simulator. Using the same score difference between a novice and an intermediate group as in a previous study on the effect of virtual reality training on laparoscopic salpingectomy [8], and using type 1 error of 0.05 and power of 0.80, needed a number of participants of 18. Thus, for our study, we recruited 20 participants.

We used SPSS 21.0–24.0 statistical software (Chicago, IL) for statistical analyses. For continuous parametric variables, we used the Independent-samples *T* test, and for non-parametric variables the Mann–Whitney U Test was used. The categorial variables were calculated by Chi-Square Tests. The reliability analysis was done by the Intraclass Correlation Coefficient test, and correlations for the parametric variables by the Pearson Correlation test and for non-parametric variables by Spearman’s rho. In analyses of learning curves, we used the Friedman test and the Wilcoxon Signed Rank Test. In the validation study, we used the Kruskal–Wallis test and Mann–Whitney U tests in post hoc analysis with Bonferroni adjustment.

The Hospital District of Helsinki and Uusimaa and the ethics committee of Helsinki University Hospital (Dnro390/13/03/03/2012) approved the study design.

## Results

**Fig. 2:**
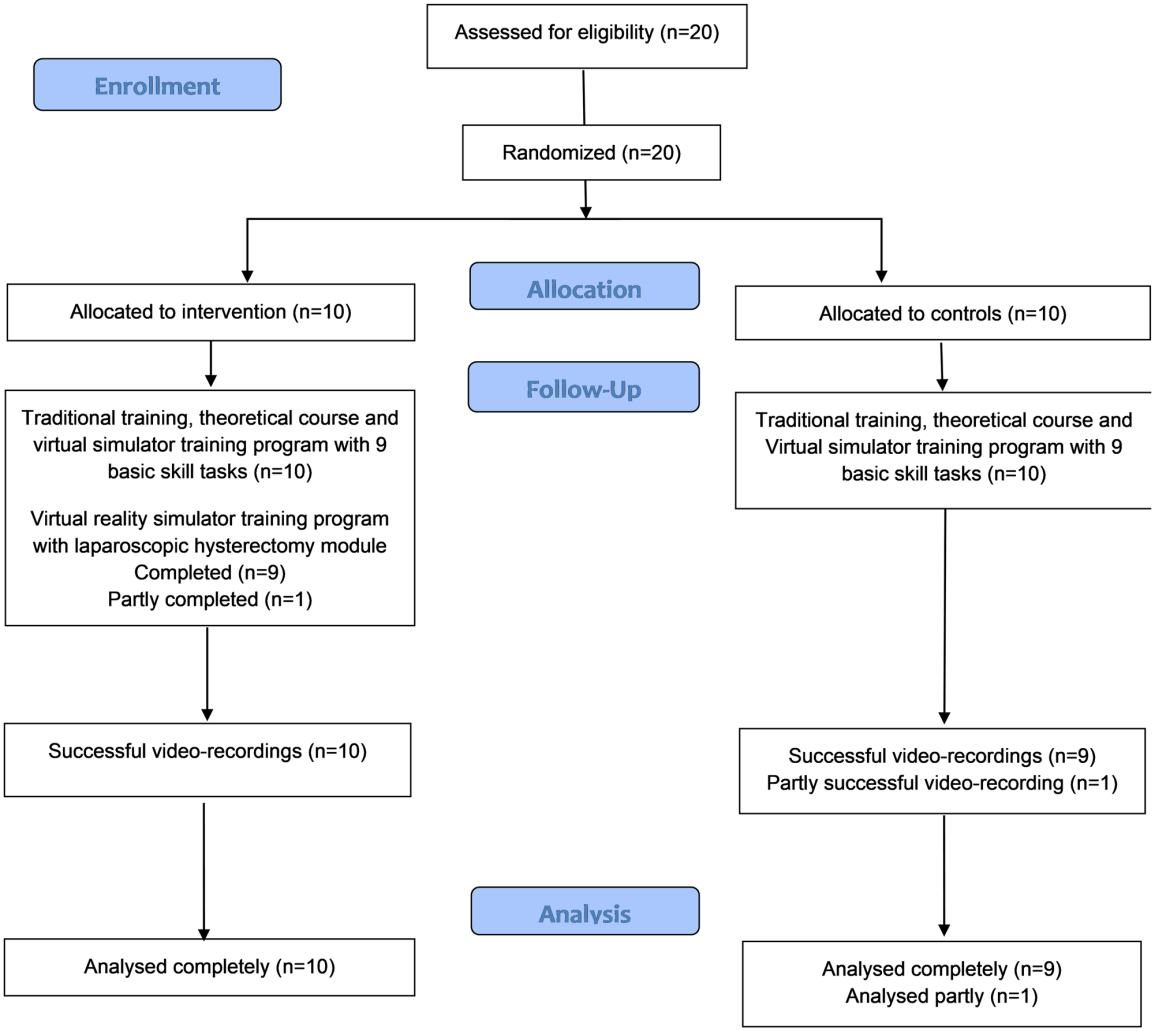
Flowchart of the participants

The flowchart is shown in Fig. 2. All but one video recording were successful; one operation was recorded only partially, and therefore was analyzed only partially. In the intervention group, nine participants of ten completed the training program as intended. One participant trained with the hysterectomy module only four times, but her operation was included in the analysis.

Demographics concerning participants, patients, and surgeries are presented in Table 1. Despite randomisation, the residents in the intervention group were more experienced in training at obstetrics and gynecology and had done more laparoscopic procedures. The composite score was higher in the control group in basic task 6 in virtual reality simulator, while in other tasks there were no differences between the groups. The overall composite score of all the tasks was higher in the control group. There was no difference in patients operated or in the size of uteri removed. In both groups, five of the patients had concomitant salpingectomy, while the rest had salpingo-oophorectomy.

**Table 1:** Operator/participant, patient, and surgery-related demographics

	Invention group	Control group	*p* value
	Mean ± standard deviation	Mean ± standard deviation	
Operator related^a^			
Age (years)	35.5 ± 2.2	31.4 ± 2.1	0.825
Experience in general Obst & Gyn (months)	38.0 ± 7.9	27.1 ± 5.7	**0.003**
Experience in general Surgery (months)	6.2 ± 0.7	6.7 ± 2.0	0.936
All previous laparoscopies done (*n*)	25.6 ± 22.5	11.8 ± 5.8	**0.027**
Basic laparoscopies done (*n*)	10.7 ± 10.9	4.9 ± 2.4	0.132
Operative laparoscopies done (*n*)	14.9 ± 13.1	6.9 ± 5.3	0.054
Video game playing (no/yes)	8/2	9/0	0.474
Musical instrument playing (no/yes)	6/3^b^	6/3	1.0
Composite scores of basic skill tasks in virtual simulator			
Task 1	99.9 ± 9.9	100.1 ± 12.5	0.974
Task 2	102.4 ± 9.5	97.3 ± 15.6	0.391
Task 3	98.3 ± 12.1	101.9 ± 11.3	0.522
Task 4	97.8 ± 8.4	102.5 ± 12.2	0.340
Task 5	101.0 ± 10.3	98.9 ± 11.5	0.624
Task 6	94.8 ± 8.6	105.8 ± 9.7	**0.009**
Task 7	96.8 ± 12.2	103.6 ± 8.6	0.185
Task 8	97.9 ± 7.2	102.3 ± 7.1	0.198
Task 9	99.7 ± 16.3	100.4 ± 8.4	0.624
Task 1–9	98.7 ± 2.3	101.4 ± 2.6	**0.033**
Patient related			
Age (years)	54.8 ± 8.7	50.2 ± 9.4	0.270
BMI (kg/m^2^)	24.9 ± 2.7	24.1 ± 2.6	0.485
Previous abdominal surgery (*n*)	0.7 ± 0.8	1.1 ± 1.2	0.466
Cesarean sections (*n*)	0	0.2 ± 0.4	0.146
Deliveries (*n*)	1.8 ± 1.2	1.0 ± 1.1	0.135
Surgery related			
Weight of the uterus (g)	185 ± 154	157 ± 123	0.660
Concomitant adnexal surgery (no/salpingectomy/salpingo-oophorectomy)	0/5/5	0/5/5	1.0
Operative time (min)	144 ± 20.8	165 ± 44.9	0.205
Blood loss (ml)	131 ± 129	121 ± 113	0.907
Direct complications (*n*)	0	1	1.0

Statistically significant (*p* < 0.05) values are highlighted in bold

^a^Data are of 19 participants

^b^Data are of nine participants

**Fig. 3:**
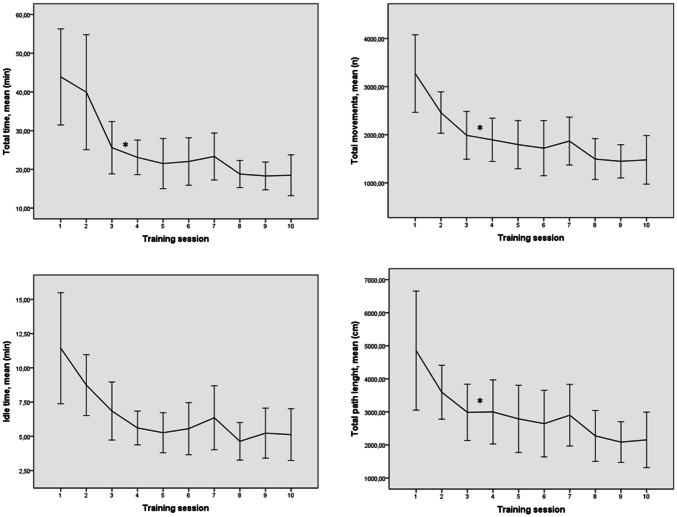
Learning curves in the hysterectomy module in virtual reality simulator. Line represents the mean and whiskers 95% confidence interval

 Learning curve plateaus were detected in total procedure time, total path length of instruments, and total number of movements of instruments (Fig. 3). In each parameter, the plateau was reached after training with the module the third time. In idle time, despite the visual plateau in the learning curve, the plateau was not detected statistically. With respect to tissue, at the first training time, dispersion of the number of events was wide, diminishing thereafter. In vascular and organ injuries, no plateaus in learning curves were detectable; the number of events per training time ranged between 0 and 11 (mean of the events 0.7–2.3) in venous and 0–5 (mean 1.2–3.5) in organ injuries.

In validation study of the procedure-specific form, the mean score for residents was 20.8 (SD 2.6), for young specialists 25.0 (SD 3.3), and for experts 27.6 (SD 6.3) (*p* = 0.01). In post hoc analyses, there was a statistically significant difference between residents and young specialists (*p* = 0.012), but not between young and experienced specialists (*p* = 0.094). Between the assessors, Cronbach’s alpha coefficient was 0.80, and the Intraclass Correlation Coefficient was 0.80 for average measures and 0.66 for single measures.

**Fig. 4:**
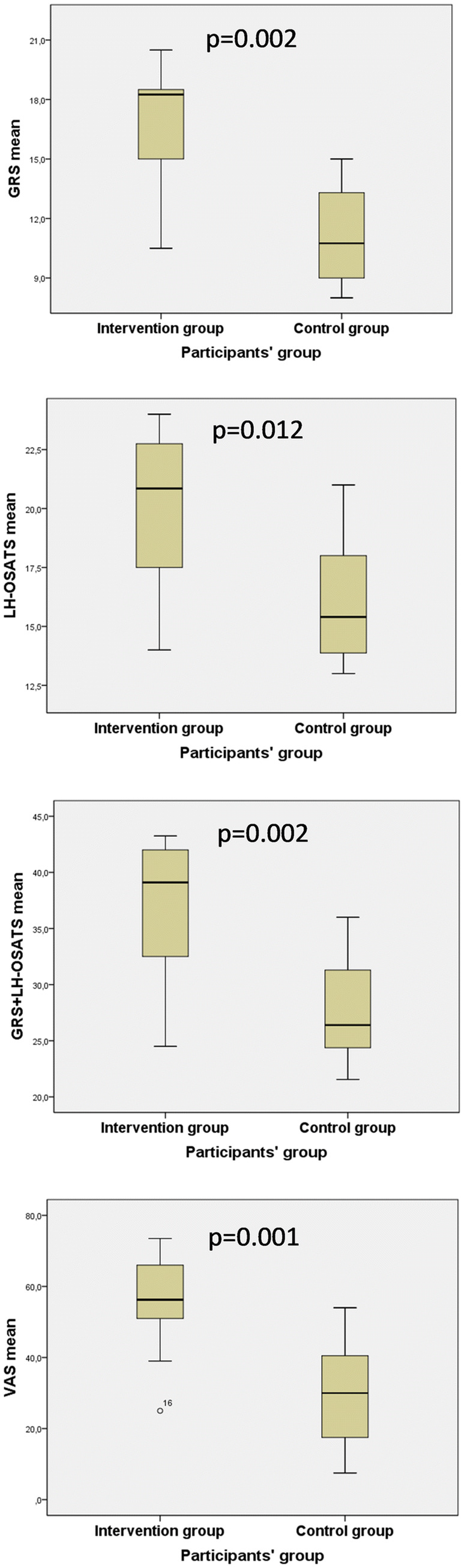
OSATS and VAS scores in the study groups. Line represents the median value, boxes 50% of the cases, and whiskers the whole range. *OSATS* objective structured assessment of technical skills. *VAS* Visual Analog Scale. *GRS* Global Rating Scale, *LH*-*OSATS* laparoscopic hysterectomy specific OSATS

The mean score in the Global Rating Scale was 17.0 (SD 3.1) in the intervention group and 11.2 (SD 2.4) in the control group (*p* = 0.002) (Fig. 4). In LH-OSATS, the mean score was 20.0 (SD 3.3) in the intervention group and 16.0 (SD 2.8) in the control group (*p* = 0.012, 95% CI 1.02–7.05). When combining both OSATS forms, the mean score was 37.0 (SD 6.2) in the intervention group and 27.5 (SD 5.2) in the control group (*p* = 0.002, 95% CI 3.96–15.12), giving Cohen’s d 1.83, meaning a large effect. In VAS, the mean score was 55.0 (SD 14.8) in the intervention group and 29.9 (SD 14.9) in the control group (*p* = 0.001, 95% CI 11.23–39.07). Between the assessors, the Intraclass Correlation Coefficient in average measures was 0.59 for GRS scores, 0.58 for LH-OSATS scores, and 0.62 for VAS, showing good reliability. Between the combined OSATS score (GRS- and LH-specific) and VAS score, we detected a strong correlation, *r* = 0.95, *p* < 0.0005.

In the intervention group, the operations required 20 min less time than in the control group (Table 1), but the difference was not statistically significant. Likewise, there was no difference in blood loss between the groups. In direct complications, there was one colon serosa lesion in the control group.

There was no correlation between hysterectomy module composite scores and scores in GRS, LH-OSATS, GRS + LH-OSATS, or VAS (correlation coefficient − 0.080, − 0.055, − 0.152, and − 0.079, respectively), neither in operative time nor in blood loss (correlation coefficient 0.305 and − 0.038, respectively).

## Discussion

In this randomized study, we showed that participants in the intervention group performed the laparoscopic hysterectomy better than the controls. Furthermore, operating time in the intervention group was 20 min shorter, although this difference was not statistically significant.

In a recent study [16], with virtual reality training curriculum for laparoscopic hysterectomy, results showed that the learning curves plateaued after 4–6 training sessions. In our study with the same parameters, plateaus in learning curves were reached already after the third training session, although the training performance improved in many parameters thereafter. At the end of the training program in our intervention group, six out of ten participants reached the set criteria. This demonstrates the importance of proficiency-based training programs instead of repetition-based. However, we were unable to show an association between the operation outcome and training program performance.

When assessing technical skills in the operating room [17], two main assessment tools have been identified: global rating scales (original or modified version of OSATS tool, or the Global Operative Assessment of Laparoscopic Skills tool, GOALS) and task-specific checklists. We used original OSATS tool for global rating skills, but we showed construct validity of a new OSATS form modified for laparoscopic hysterectomy. Specific tools for assessing competence in laparoscopic supracervical hysterectomy [18] and total laparoscopic hysterectomy [19] have been published, but we chose to use a more general form concentrating on the core steps in laparoscopic hysterectomy. Quite recently, a form for laparoscopic hysterectomy [20] was published, but unfortunately this form was not available at the time our study was ongoing.

The VAS score is typically used to assess pain or anxiety among patients, but it can be also used for other purposes, e.g., among residents for assessing their own management in a special kind of anesthesia [21], and in assessing the overall quality of patient sign-out from the emergency department [22]. For trainees’ surgical skills, the VAS score has been used to evaluate suturing and knot tying skills [23], showing the VAS score and the OSATS for global rating skills ‘good’ for educational purposes with interrater reliability (IRR) 0.71 in a group where assessors were trained to the use of scales. The IRR was slightly lower, though VAS scores correlated well with the combined OSATS score, and the scale was easy to use. In our study, we used young specialist’s level as a reference instead of an expert’s level. This allowed us to use wider distribution in scores.

The strengths of our study include well-documented practice session programs with the virtual reality simulator. Also, all participants did an online learning module as a web course ‘Basics in gynecological laparoscopy’ [12] and passed the online test. This type of cognitive training has been shown to transfer practical skills in the operating room [24]. We also used live operations to assess the study outcomes and video recordings for accurate and blind evaluation. Furthermore, the assessors were blinded for the operator and his/her study group, and the IRR between assessors was good. In addition to OSATS-GRS and VAS scales, we used a procedure-specific form to assess the operations more accurately. We showed construct validity of this new assessment tool for investigational purposes, but full validation and routine use of the form would require further studies with other aspects of validity and cost implications.

Our study also has limitations. First, the recruitment time was long, however, the standard clinical education was not altered during the study. Participation in the study was voluntary, but since all suitable residents participated, potential recruitment bias was avoided. Second, the sample size was small and by chance, the participants in the intervention group had longer experience in obstetrics and gynecology and they had done more operative laparoscopies than the control group. However, in the beginning of the study they were not more competent in the basic skills in the virtual reality simulator, but the longer working period in the operating room could have caused an advantage in operating as a first surgeon. Third, the intervention consisted of training hysterectomy module in virtual reality simulator offering only limited realism. Every participant had been assisting in more advanced laparoscopies, and online material included information on performing a laparoscopic hysterectomy. Thus, our aim was to evaluate the value of repeated procedural training with virtual reality simulator targeting on learning and automatization of the surgical steps needed. And finally, the impact of the assistant during a laparoscopic hysterectomy is difficult to standardize and evaluate. It is obvious that residents need guidance while performing their first laparoscopic hysterectomy. The instrument of the assisting doctor is clearly visible in the videos, and hence the actions of the senior doctor is possible to note and exclude while evaluating the steps of the operation. When evaluating GRS and VAS scores, only the actions of the junior doctor were taken into consideration. Moreover, the different senior doctors as assistants were randomly assigned and thus, likely, the significance of the assistance is equal in both groups.

To conclude, we found a significant increase in OSATS and VAS scores in live laparoscopic hysterectomies after training with a laparoscopic hysterectomy module on a virtual reality simulator. This indicates that skills gained in the virtual reality simulator seems to be transferred into the operating room, and training with a virtual reality simulator may lead to better surgical outcomes. While the training program on the virtual reality simulator hysterectomy module is relatively easy to implement, including it into the laparoscopic hysterectomy training curriculum could be considered.

## References

[1] Elbadrawy M, Majoko F, Gasson J (2008). Impact of Calman system and recent reforms on surgical training in gynaecology. J Obstet Gynaecol.

[2] Vitish-Sharma P, Knowles J, Patel B (2011). Acquisition of fundamental laparoscopic skills: is a box really as good as a virtual reality trainer?. Int J Surg.

[3] Mettler LL, Dewan P (2009). Virtual reality simulators in gynecological endoscopy: a surging new wave. JSLS.

[4] Beyer-Berjot L, Aggarwal R (2013). Toward technology-supported surgical training: the potential of virtual simulators in laparoscopic surgery. Scand J Surg.

[5] Li L, Yu F, Shi D, Shi J, Tian Z, Yang J (2017). Application of virtual reality technology in clinical medicine. Am J Transl Res.

[6] Grantcharov TP, Reznick RK (2008). Teaching procedural skills. BMJ.

[7] Grantcharov TP, Kristiansen VB, Bendix J, Bardram L, Rosenberg J, Funch-Jensen P (2004). Randomized clinical trial of virtual reality simulation for laparoscopic skills training. Br J Surg.

[8] Larsen CR, Soerensen JL, Grantcharov TP, Dalsgaard T, Schouenborg L, Ottosen C (2009). Effect of virtual reality training on laparoscopic surgery: randomised controlled trial. BMJ.

[9] Thomsen AS, Bach-Holm D, Kjaerbo H, Hojgaard-Olsen K, Subhi Y, Saleh GM (2017). Operating room performance improves after proficiency-based virtual reality cataract surgery training. Ophthalmology.

[10] Yiannakopoulou E, Nikiteas N, Perrea D, Tsigris C (2015). Virtual reality simulators and training in laparoscopic surgery. Int J Surg.

[11] Garry R (2005). The future of hysterectomy. BJOG.

[12] Jokinen E, Mikkola TS, Harkki P (2017). Evaluation of a web course on the basics of gynecological laparoscopy in resident training. J Surg Educ.

[13] Jokinen E, Mikkola TS, Harkki P (2019). Effect of structural training on surgical outcomes of residents’ first operative laparoscopy: a randomized controlled trial. Surg Endosc.

[14] Rosenthal R, von Websky MW, Hoffmann H, Vitz M, Hahnloser D, Bucher HC (2015). How to report multiple outcome metrics in virtual reality simulation. Eur Surg.

[15] Martin JA, Regehr G, Reznick R, MacRae H, Murnaghan J, Hutchison C (1997). Objective structured assessment of technical skill (OSATS) for surgical residents. Br J Surg.

[16] Crochet P, Aggarwal R, Knight S, Berdah S, Boubli L, Agostini A (2017). Development of an evidence-based training program for laparoscopic hysterectomy on a virtual reality simulator. Surg Endosc.

[17] Fahim C, Wagner N, Nousiainen MT, Sonnadara R (2018). Assessment of technical skills competence in the operating room: a systematic and scoping review. Acad Med.

[18] Goderstad JM, Sandvik L, Fosse E, Lieng M (2016). Assessment of surgical competence: development and validation of rating scales used for laparoscopic supracervical hysterectomy. J Surg Educ.

[19] Tremblay C, Grantcharov T, Urquia ML, Satkunaratnam A (2014). Assessment tool for total laparoscopic hysterectomy: a Delphi consensus survey among international experts. J Obstet Gynaecol Can.

[20] Knight S, Aggarwal R, Agostini A, Loundou A, Berdah S, Crochet P (2018). Development of an objective assessment tool for total laparoscopic hysterectomy: a Delphi method among experts and evaluation on a virtual reality simulator. PLoS ONE.

[21] Bilotta F, Titi L, Lanni F, Stazi E, Rosa G (2013). Training anesthesiology residents in providing anesthesia for awake craniotomy: learning curves and estimate of needed case load. J Clin Anesth.

[22] Milano A, Stankewicz H, Stoltzfus J, Salen P (2019). The impact of a standardized checklist on transition of care during emergency department resident physician change of shift. West J Emerg Med.

[23] Robertson RL, Vergis A, Gillman LM, Park J (2018). Effect of rater training on the reliability of technical skill assessments: a randomized controlled trial. Can J Surg.

[24] Jayakumar N, Brunckhorst O, Dasgupta P, Khan MS, Ahmed K (2015). e-Learning in surgical education: a systematic review. J Surg Educ.

